# Supplementary material for: VAX014, an Oncolytic Therapy, Reduces Adenomas and Modifies Colon Microenvironment in Mouse Model of CRC
Source: Int J Mol Sci. 2023 Jun 10;24(12):9993. doi: 10.3390/ijms24129993 (PMC10298379; doi:10.3390/ijms24129993)
Supplement: Supplementary file 1 [file ijms-24-09993-s001.zip › Grenier et al. Supplemental Files/ijms-2388865 supplementary.pdf]

Supplementary Materials

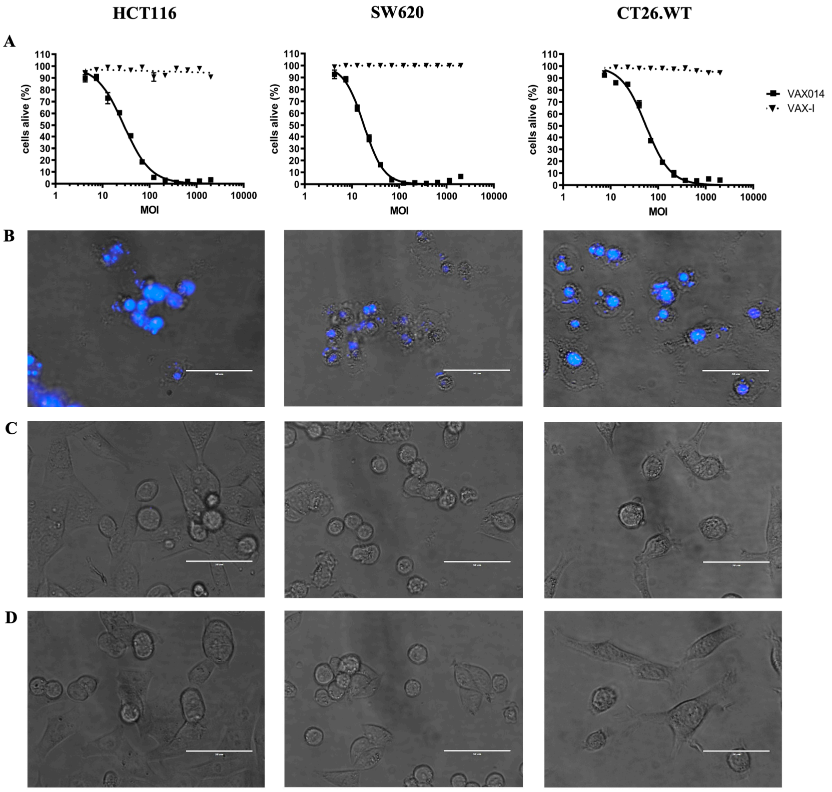

Supplemental Figure S1. Viability of HCT116, SW620, and CT26.WT CRC cell lines (**A**) was measured using Prestoblue™ viability dye after 20 hours of coincubation with increasing MOI concentrations of VAX014 and VAX-I rBMCs to mammalian cells. The ability of VAX014 to induce nuclear membrane permeabilization in the same CRC cell lines treated with VAX014 (**B**) VAX-I (**C**) or negative control (**D**) was assessed using DAPI staining after 20 hours of coincubation (scale bar (white) 50 µm).

Formatted: Not Highlight

Formatted: Not Highlight

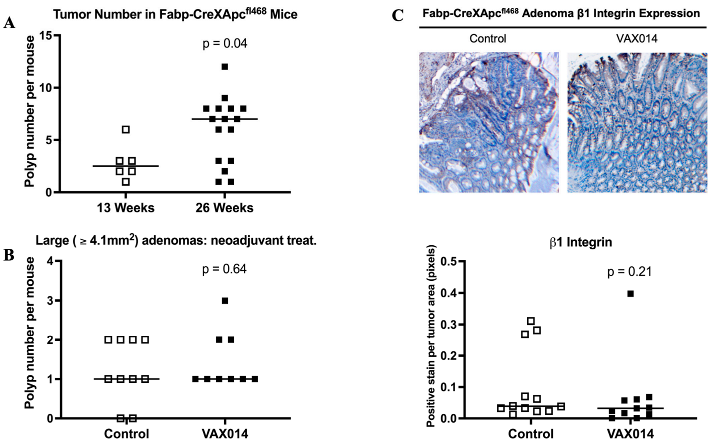

Supplemental Figure S2. Macroscopic adenoma number quantification of naïve Fabp-CreXApc^{fl468} mice (**A**) at 14 weeks and 26 weeks of age (14 weeks n=6, 26 weeks n=16). (**B**) The abundance of large, ≥ 4 mm^2, adenomas were investigated in Fabp-CreXApc^{fl468} mice at 26 weeks after weekly intrarectal neoadjuvant treatment (14-19 weeks) (VAX014 n=9, Control n=10). IHC analysis (5X magnification) of β1 integrin (**C**) expression in adenomas harvested from Fabp-CreXApc^{fl468} mice treated with VAX014 from 22-24 weeks of age and sacrificed 1 hour after final treatment. Median values are represented on graphs. Statistical significance was assessed by the Mann-Whitney U Test.

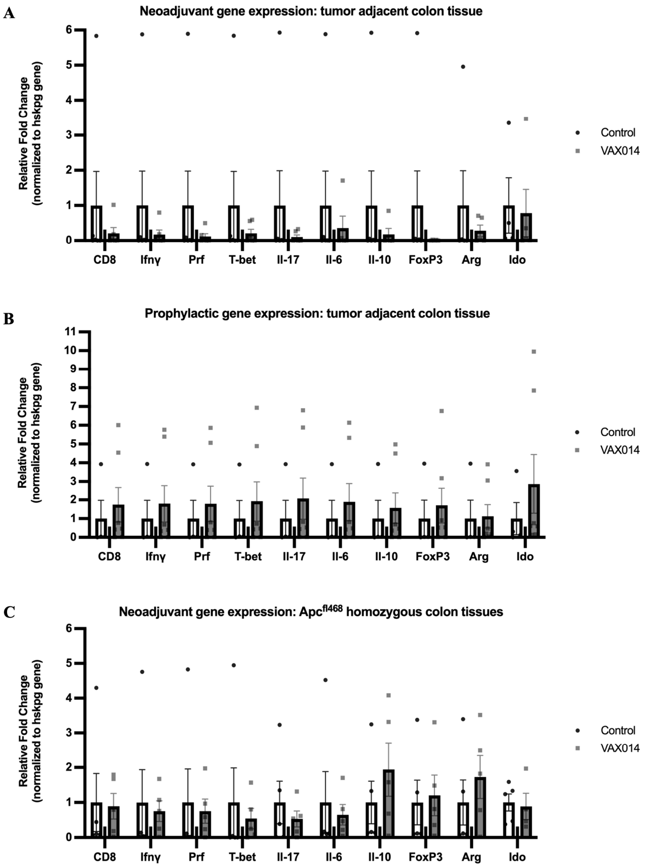

Supplemental Figure S3. A gene panel of cytokine markers associated with inflammation, anti-tumor immune responses and immune suppression were analyzed in tumor adjacent, i.e normal tissues directly adjacent to adenomas, colon tissues harvested from neoadjuvant (VAX014 n=6, Control n=6) (14-19 week) (**A**) and prophylactic (8-13 week) (VAX014 n=7, Control n=4) (**B**) VAX014 treatment groups at 26-weeks-old. Immune gene markers were also analyzed in 26-week-old parental homozygous Apc^{fl468} mice (VAX014 n=5, Control n=5) (**C**) which do not develop adenomas and were treated with VAX014 from 14-19 weeks. Means with SEM are displayed; the Mann-Whitney U test was used to evaluate significance. Control (white), VAX014 (grey). * $p < 0.05$.

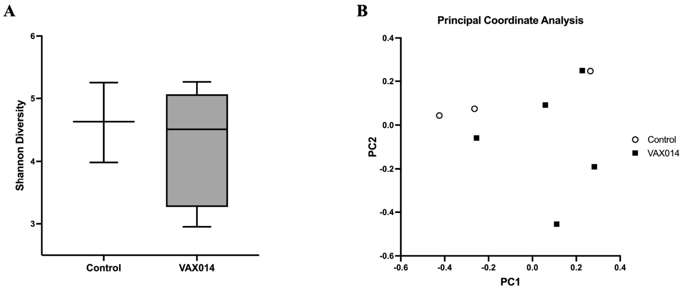

Supplemental Figure S4. Diversity analysis of microbiomes isolated from tissue adjacent tissues isolated from 26-week-old mice with neoadjuvant VAX014 treatment (VAX014 n=5, Control n=3) using the Shannon diversity index to assess alpha diversity (**A**) and Principal coordinate analysis derived from weighted UniFrac beta diversity distance values (**B**). Statistical significance was analyzed using the Mann-Whitney U test for Shannon diversity analysis and Anosim for Principal coordinate analysis.

Table S1. Summary of immune marker expression in FABP-CreXApc^{fl468} treated with VAX014

Table S2. Sequences of primers used for Real-time PCR

File S1. Taxonomic analysis of FABP-CreXApc^{fl468} colonic microbiomes treated with VAX014

File S2. Gene function analysis of FABP-CreXApc^{fl468} colonic microbiomes treated with VAX014
